# The red seaweed *Plocamium brasiliense* shows anti-snake venom toxic effects

**DOI:** 10.1186/s40409-015-0002-2

**Published:** 2015-02-10

**Authors:** Geisiane Alves da Silva, Thaisa Francielle Souza Domingos, Rainiomar Raimundo Fonseca, Eladio Flores Sanchez, Valéria Laneuville Teixeira, André Lopes Fuly

**Affiliations:** Department of Molecular and Cellular Biology, Institute of Biology, Federal Fluminense University (UFF), Niterói, Rio de Janeiro State Brazil; Department of Organic Chemistry, Institute of Chemistry, Federal Fluminense University (UFF), Niterói, Rio de Janeiro State Brazil; Laboratory of Biochemistry of Proteins from Animal Venoms, Research and Development Center, Ezequiel Dias Foundation, Belo Horizonte, Minas Gerais State Brazil; Department of Marine Biology, Institute of Biology, Federal Fluminense University (UFF), Niterói, Rio de Janeiro State Brazil

**Keywords:** *Bothrops jararaca*, Snake venom, *Plocamium brasiliense*, Seaweed, Antivenom, Bioprospecting

## Abstract

**Background:**

Snakebite is considered a neglected tropical disease by the World Health Organization. In Brazil, about 70% of the envenomation cases are caused by *Bothrops* snakes. Its venom may provoke hemorrhage, pain, necrosis, hemolysis, renal or cardiac failure and even death in victims. Since commercial antivenom does not efficiently neutralize the local toxic effects of venoms, natural products have been tested in order to provide alternative or complementary treatment to serum therapy. Therefore, the present study aimed to evaluate the ability of the seaweed *Plocamium brasiliense* and its active derivatives to neutralize hemorrhagic, edematogenic, hemolytic, coagulant and proteolytic activities of *B. jararaca* venom.

**Methods:**

Specimens of *P. brasiliense* were collected in Rio de Janeiro state, Brazil, dried and submitted to oil extraction using four solvents of increasing polarities, n-hexane (HEX), dichloromethane (DCM), ethyl acetate (ETA) and hydroalcoholic solution (HYD). The solvents were evaporated, yielding HEX, DCM, ETA and HYD extracts. Further, all extracts were dissolved in dimethylsulfoxide. In addition, two monoterpenes (8-bromo-3,4,7-trichloro-3,7-dimethyl-1E, 5E-octadiene and 1,8-dibromo-3,4,7-trichloro-3,7-dimethyl-1E, 5E-octadiene) and a cholesterol fraction were isolated from the extract of *P. brasiliense* prepared in hexane. Algal samples were incubated for 30 minutes with *B. jararaca* venom, and then tested for lethality; hemorrhagic, edematogenic, hemolytic, coagulant and proteolytic effects.

**Results:**

Most of the algal extracts inhibited the toxic effects with different potencies. The DCM extract was the most effective, since it inhibited all types of toxic activity. On the other hand, the HYD extract failed to inhibit any effect. Moreover, the isolated products inhibited proteolysis and protected mice from hemorrhage in 30% of the cases, whereas 8-bromo-3,4,7-trichloro-3,7-dimethyl-1E, 5E-octadiene inhibited 100% and 20% of the hemorrhagic and proteolytic activities, respectively. None of the algal products were toxic to mice.

**Conclusion:**

Seaweeds may be a promising source of inhibitors against toxic effects caused by *B. jararaca* envenomation, which may contribute to antivenom treatment.

## Background

Snakebites comprise a serious health problem in numerous parts of the world, due to their seriousness, sequelae and high fatality rates. According to the World Health Organization [1], up to five million people are bitten by snakes every year, and around 2 million of these people die, whereas 400,000 cases end in amputations and other severe health consequences, such as infection, tetanus, scarring, contractures, and psychological sequelae [1,2]. According to Kasturiratne *et al.* [3], 420,000 to 1,841,000 snakebites occur per year worldwide with 20,000 to 94,000 deaths. However, research indicates that the burden of envenomation by snakes may be even heavier.

In Brazil, snakes of the *Bothrops* genus are involved in most cases of human envenomation, about 70%, particularly the species *B. jararaca* [4,5]. *Bothrops* venoms, which have been extensively studied, are composed of a complex mixture of proteins, peptides and other organic and inorganic molecules that induce local (pain, edema, necrosis, inflammation, hemorrhage) and systemic (coagulation disturbances, renal and cardiac failure) effects in victims [6-13]. *B. jararaca* is found in southern Brazil, Paraguay, and northern Argentina. Envenomation by this species has a similar profile to that observed in other *Bothrops* species including shock, tissue necrosis, intravascular coagulation, local and systemic hemorrhage and edema.

The recommended treatment for envenomation by this species is polyvalent *Bothrops* antivenom, which is produced by horse hyperimmunization [14-16]. In spite of inhibiting systemic toxic effects, antivenom has a few disadvantages including ineffectiveness against local effects and side effects [17-19]. In addition, the variability in venom composition of different *Bothrops* species may affect the antivenom efficacy. Therefore, alternative strategies and treatments have been extensively investigated [20,21]. In most developing countries, about 80% of snakebite victims seek help first from traditional practitioners that use plant extracts. Appropriate medical care with antivenom therapy is usually their second option [21]. Thus, natural products are good candidates for alternative treatments. The seas provide an amazing source of molecules when compared to the terrestrial environment; however, little is known about their biological or pharmacological functions. Marine organisms produce molecules derived from primary and secondary metabolism with biological and pharmacological effects, such as antiviral, anticancer, anticoagulant and antiplatelet activities [22-26]. Previously, antivenom activity has been described in marine algae and sponges, which showed that both organisms can inhibit some toxic effects of *Lachesis muta* and *B. jararaca* venoms [27,28].

**Figure 1:**
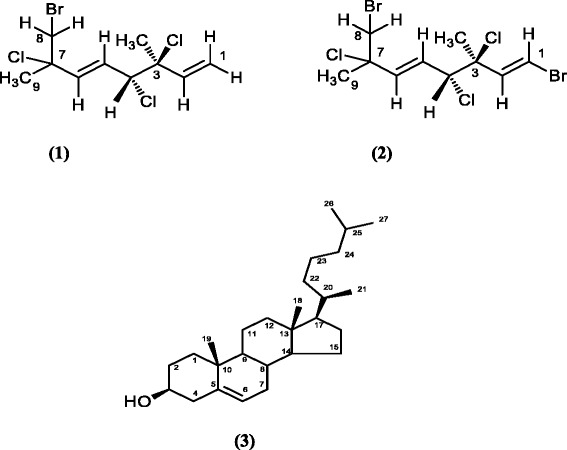
**Chemical structure of (1) monoterpene 1 (8-bromo-3,4,7-trichloro-3,7-dimethyl-1E,5E-octadiene), (2) monoterpene 2 (1,8-dibromo-3,4,7-trichloro-3,7-dimethyl-1E,5E-octadiene) and of the (3) cholesterol fraction.**

In the present study, the red seaweed *Plocamium brasiliense* (Greville) M. Howe and W.R. Taylor has been investigated. The genus *Plocamium* Lamouroux (Plocamiaceae, Rhodophiceae) contains more than 40 species that are widely distributed over the oceans. *P. brasiliense* is abundant off the east-southeast coast of Brazil [29]. These marine algae produce acyclic and cyclic halogenated monoterpenes, of which more than 100 molecules have been isolated [29,30]. Such molecules have shown biological activities including antimicrobial, antifungal, ichthyotoxic, cytotoxic and insecticidal properties as well as antiviral activity against herpes virus [31,32]. Moreover, *P. brasiliense* may be used as herbicide or even as food [33]. It has been shown that three products isolated from *P. brasiliense* inhibited some toxic *in vitro* and *in vivo* activities of *B. jararaca* venom: monoterpene 1 (8-bromo-3,4,7-trichloro-3,7-dimethyl-1E, 5E-octadiene), monoterpene 2 (1,8-dibromo-3,4,7-trichloro-3,7-dimethyl-1E, 5E-octadiene) and a cholesterol [30,32,34]. Their chemical structures are shown in Figure 1.

The aim of the present study was to evaluate the inhibitory potential of extracts of *P. brasiliense* and of their isolated products (the two monoterpenes and cholesterol) against some *in vitro* (proteolysis, coagulation and hemolysis) and *in vivo* (hemorrhage, lethality and edema) toxic effects induced by *B. jararaca* snake venom.

## Methods

### Algal material

Specimens of *P. brasiliense* were collected by snorkeling in October 2010, at Enseada do Forno beach, in the city of Armação de Búzios, located in the north of Rio de Janeiro state, Brazil (22° 45′ S, 41° 52′ W). They were washed with local seawater and separated from sediments, epiphytes and other associated organisms. The algae were dried for twenty days and their extracts were obtained by using appropriate solvent. Voucher specimens were deposited in the Herbarium of the Rio de Janeiro State University (HRJ 10331–32). The Brazilian algal collection license (VLT) number is 10594 (IBAMA/SISBIO).

### Preparation of crude extracts and fractions

*P. brasiliense* was collected and dried at room temperature (average of 28°C). Then, the algal crude extract was ground in an industrial blender and placed into a plastic tray, yielding 5.16 g of powdered algae. *P. brasiliense* oil extraction was performed using the solvents hexane, dichloromethane, ethyl acetate and ethanol/water. Afterwards, all solvents were evaporated off under reduced pressure, yielding a crude residue. An aliquot of each extract was weighed, aliquoted and frozen at −20°C, and finally, the extracts were dissolved in dimethylsulfoxide (DMSO, 30% v/v) to perform the biological assays.

The extracts were obtained according to Fonseca *et al.* [31] and yielded the following values: n-hexane, 500 mg; dichloromethane, 196 mg; ethyl acetate, 42 mg; and ethanol/water, 250 mg. The extract prepared in n-hexane (500 mg) was subjected to silica gel 70–230 mesh column chromatography (4 × 70 cm) eluted with n-hexane, CH_2_Cl_2_, EtOAc and MeOH in sequence, resulting in 97 fractions of 10 mL each (F1 to F97) and 55 fractions of 20 mL each (F98 to F153). The fraction F17 (18 mg) eluted with n-hexane/CH_2_Cl_2_ (9.7:0.3) and the fraction F76 (16 mg) eluted with n-hexane/CH_2_Cl_2_ (5:5) contained the pure halogenated monoterpenes 1 and 2, respectively. The fractions F112 to F118 eluted with n-hexane/CH2Cl2 (3.5:6.5) and F119-F125 eluted with n-hexane/CH_2_Cl_2_ (3:7) contained the pure cholesterol (42 mg). These fractions were also obtained according to the method by Fonseca *et al.* [31], and were analyzed according to Vasconcelos *et al.* [30] by HRGC/MS on a HP 5890 series GC system, coupled to a HP 5973 mass selective detector in the EI mode (70 eV) equipped with a HP-1 MS capillary column (30 m × 0.25 mm, film thickness 0.25 μm), and dissolved in DMSO (30%, v/v) as well.

### Venom and animals

*B. jararaca* venom was kindly supplied by the Ezequiel Dias Foundation (FUNED), Belo Horizonte, Minas Gerais state, Brazil, vacuum dried and stored at −20°C until use. Male Balb/c mice (*Mus musculus* species) weighting 18–20 g were obtained from the Center of Laboratory Animals (NAL) of the Federal Fluminense University (UFF). They were housed under controlled conditions of temperature (24 ± 1°C) and light. All the experiments performed were approved by the UFF Institutional Committee for Ethics in Animal Experimentation (CEUA: 200/10) and were in accordance with the guidelines of the Brazilian Committee for Animal Experimentation (COBEA).

### Antihemolytic activity

The degree of hemolysis of *B. jararaca* venom was determined by the indirect hemolytic test using human erythrocytes and hen’s egg yolk emulsion as substrates [35]. The lowest amount of *B. jararaca* venom that produced 100% hemolysis was called the minimum indirect hemolytic dose (MIHD). Inhibitory experiments were performed by incubating algal extracts with one MIHD for 30 minutes at room temperature, and then, hemolytic activity was evaluated. Positive controls were performed by incubating venom with DMSO (5% v/v) or saline solution, instead of extracts. DMSO (5% v/v) or extracts alone were used as negative controls.

### Anticoagulant activity

The coagulant activity of *B. jararaca* venom was monitored using a digital Amelung coagulometer, model KC4A (Labcon, Germany). Different concentrations of *B. jararaca* venom were mixed with diluted citrated plasma (1:1 in saline) donated from healthy volunteers collected at a local public blood bank (University Hospital Antônio Pedro of UFF). The amount of venom (μg/mL) that clotted plasma in 60 seconds was called the minimum coagulant dose (MCD). To evaluate the inhibitory effect, algal extracts were incubated for 30 minutes at room temperature with one MCD, and then, the mixture was added to plasma and coagulation time was recorded. Positive controls were performed in parallel by incubating venom with DMSO (1% v/v) or saline solution. DMSO (1% v/v) or extracts alone were used as negative controls.

### Antiproteolytic activity

Proteolytic activity of *B. jararaca* venom was determined using azocasein as substrate (0.2% w/v, in 20 mM Tris–HCl, 8 mM CaCl_2_, pH 8.8) [36] – with minor modifications. An effective concentration (EC) was defined as the amount of *B. jararaca* venom (μg/mL) able to produce a variation of about 0.2 OD units at A 420 nm (spectrophotometer Hitachi U-5100). The inhibitory effect of algal extracts was performed by incubating them with two EC of *B. jararaca* venom for 30 minutes at room temperature and then, proteolysis was measured. Similarly, control experiments were conducted by mixing venom with DMSO (5% v/v) or saline solution and, further proteolysis was performed. DMSO (5% v/v) or extracts were used as negative controls.

### Antihemorrhagic activity

Hemorrhagic lesions produced by *B. jararaca* venom were quantified using a procedure described by Kondo *et al.* [37] with modifications. Briefly, samples (100 μL) were injected intradermally (i.d.) into abdominal skin of mice. Two hours later, the animals were euthanized, abdominal skin was removed, stretched and inspected for visual changes in the inner surface of subcutaneous layers in order to find hemorrhagic spots. Hemorrhage was classified into minimum hemorrhagic dose (MHD), defined as the amount of *B. jararaca* venom (μg/g) able to produce a hemorrhagic halo of 10 mm [38]. The inhibitory effect of algal extracts was investigated by incubating them with two MHD of *B. jararaca* venom for 30 minutes at room temperature and then, the mixture (100 μL) was injected i.d. into mice and hemorrhage was observed. Hemorrhagic activity was expressed as the mean diameter (in millimeters) of the hemorrhagic halo induced by *B. jararaca* venom in the absence and presence of algae. Negative controls were performed by injecting only DMSO (5% v/v), extracts or saline solution.

### Antiedematogenic activity

Edema-inducing activity of *B. jararaca* venom was determined according to Yamakawa *et al.* [39]. Groups of five mice received 50 μL of *B. jararaca* venom subcutaneously (s.c.) in the right paw, while the left paw received 50 μL of saline solution or DMSO. One hour after injection, edema was evaluated as the percentage increase in weight of the right paw compared to the left one. Antiedematogenic activity was performed by incubating algal extracts with *B. jararaca* venom for 30 minutes at room temperature, and then the mixture was injected s.c. into mice. Control experiments were performed by mixing *B. jararaca* venom with DMSO (5% v/v) or saline solution. For negative controls, DMSO (5% v/v) or extracts were injected.

### Anti-lethal activity

Groups of six mice received (100 μL) intraperitoneally (i.p.) *B. jararaca* venom (20 μg/g) and were observed for 48 hours. The anti-lethal assay was performed by incubating *B. jararaca* (20 μg/g) with algal extracts (80 μg/g) for 30 minutes at room temperature and the mixture was injected i.p. into mice. Positive control groups received *B. jararaca* mixed with saline solution or DMSO, and negative controls received algal extracts instead of venom.

### Statistical analysis

Results are presented as means ± S.E.M. The statistical significance of differences between tests was evaluated by Student’s unpaired *t*-test. p values of < 0.05 were considered statistically significant.

## Results

### Effect of *P. brasiliense* extracts on *B. jararaca* venom-induced coagulation

**Figure 2:**
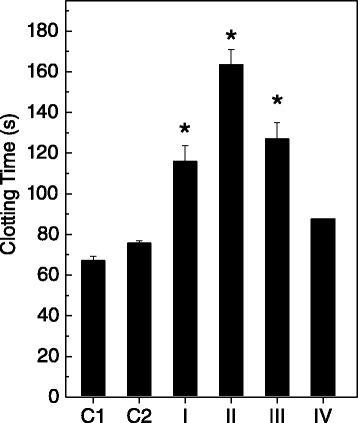
**Effect of** ***P. brasiliense*** **on plasma coagulation of** ***B. jararaca*** **venom.** The venom (20 μg/mL) was incubated with algal extracts (100 μg/mL) prepared in n-hexane (group I), dichloromethane (group II), ethyl acetate (group III) or hydroalcoholic solution (group IV), then the mixtures were added to plasma and coagulation was monitored. Control groups consisted of venom incubated with NaCl (C1) or with DMSO (C2). Results are expressed as mean ± SE of two individual experiments (n = 3). *p < 0.05 when compared to C1 or C2.

*B. jararaca* venom (20 μg/mL) clots plasma usually in 70 seconds. Therefore, this venom concentration was mixed with *P. brasiliense* extracts (100 μg/mL) in order to evaluate the effects on coagulation (Figure 2). As shown in Figure 2, the algal extracts inhibited the coagulant activity of *B. jararaca* venom in different degrees. The extracts prepared in dichloromethane (Figure 2, group II) and hydroalcoholic solution (Figure 2, group IV) inhibited more and less efficiently, respectively. When tested alone, the extracts did not induce coagulation.

### Effect of *P. brasiliense* extracts on *B. jararaca* venom-induced proteolysis

**Figure 3:**
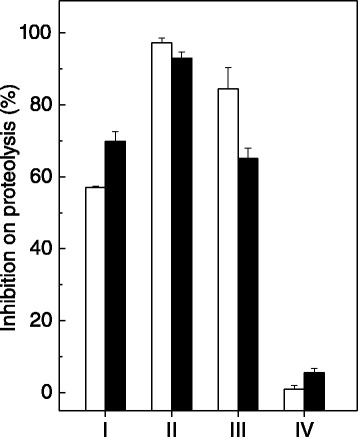
**Effect of** ***P. brasiliense*** **extracts on proteolysis induced by** ***B. jararaca*** **venom.** *P. brasiliense* extracts (10 μg/mL, white columns) and (100 μg/mL, black columns) prepared in n-hexane (group I), dichloromethane (group II), ethyl acetate (group III) or hydroalcoholic solution (group IV) were incubated with *B. jararaca* venom (10 μg/mL). Results are expressed as mean ± SE of two individual experiments (n = 3).

*P. brasiliense* extracts (10 and 100 μg/mL) inhibited the proteolytic activity of 10 μg/mL of *B. jararaca* venom (Figure 3). Regardless of the concentration, the dichloromethane extract had the highest inhibitory activity (Figure 3, group II), while the hydroalcoholic solution did not inhibit at all (Figure 3, group IV). The other two extracts (n-hexane, group I, and ethyl acetate, group III, at 100 μg/mL) inhibited proteolysis by 57% and 84%, respectively. In addition, the monoterpenes 1 and 2 inhibited by 20% and 38%, respectively, while the cholesterol inhibited proteolysis induced by *B. jararaca* venom by 32% (Table 1). The extracts alone were not able to hydrolyze azocasein.

**Table 1:** **Antiproteolytic and antihemorrhagic effects of** ***P. brasiliense*** **products**

**Sample**	**Inhibition (%)**	
	**Proteolysis**	**Hemorrhage**
*B. jararaca* + monoterpene 1	20 ± 4.5	100
*B. jararaca* + monoterpene 2	37 ± 2.1	32 ± 11
*B. jararaca* + cholesterol	33 ± 2.5	28 ± 6

Monoterpene 1: 8-bromo-3,4,7-trichloro-3,7-dimethyl-1E,5E-octadiene; monoterpene 2: 1,8-dibromo-3,4,7-trichloro-3,7-dimethyl-1E,5E-octadiene.

### Effect of *P. brasiliense* extracts on *B. jararaca* venom-induced hemolysis

**Figure 4:**
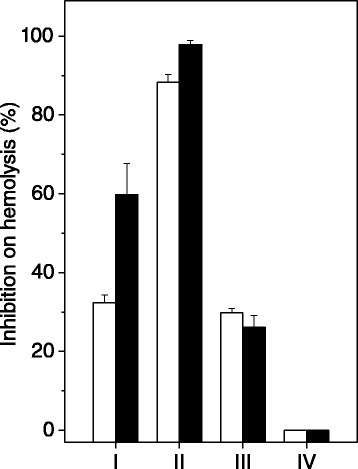
**Effect of** ***P. brasiliense*** **extracts on hemolysis provoked by** ***B. jararaca*** **venom.** Different concentrations of *P. brasiliense* algal extracts (90 μg/mL, white columns or 180 μg/mL, black columns) prepared in n-hexane (group I), dichloromethane (group II), ethyl acetate (group III) or hydroalcoholic solution (group IV) were incubated with *B. jararaca* venom (30 μg/mL). Results are expressed as mean ± SE of two individuals experiments (n = 3).

The extracts were not able to lyse erythrocytes. However, the hemolysis caused by *B. jararaca* venom (30 μg/mL) was inhibited significantly by all *P. brasiliense* extracts (90 and 180 μg/mL), except for the hydroalcoholic solution (Figure 4). The extract prepared in dichloromethane inhibited hemolysis by 88% and 98%, at 1:3 and 1:6 venom:algae ratios (w/w), respectively (Figure 4, group II). At both venom:algae ratios, the ethyl acetate extract inhibited proteolysis by 25% (Figure 4, group III).

### Effect of *P. brasiliense* extracts on *B. jararaca* venom-induced hemorrhage, edema and lethality

**Figure 5:**
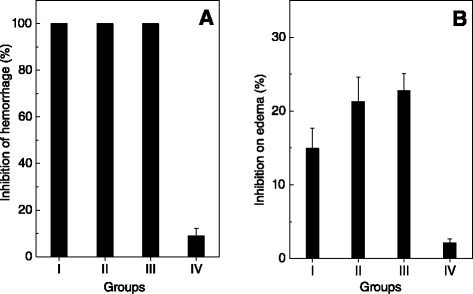
**Effect of** ***P. brasiliense*** **extracts on hemorrhage and edema induced by** ***B. jararaca*** **venom.** The algal extracts (35 μg/g) prepared in n-hexane (group I), dichloromethane (group II), ethyl acetate (group III) and hydroalcoholic solution (group IV) were incubated with *B. jararaca* venom (25 μg/g). Then, mixtures were injected into mice and hemorrhagic **(Panel A)** or edematogenic **(Panel B)** activities were analyzed, as described in methods. Results are expressed as mean ± SE of two individuals experiments (n = 3).

*P. brasiliense* extracts prepared in different solvents and their isolated products inhibited hemorrhage and edematogenic activities *in vivo* of *B. jararaca* venom (Figure 5). The extracts (15 μg/mL) prepared in n-hexane (group I), dichloromethane (group II) and ethyl acetate (group III) fully inhibited *B. jararaca* venom-induced hemorrhage, except for the one prepared in hydroalcoholic solution (group IV) (Figure 5A). The isolated products from *P. brasiliense* (15 μg/mL) monoterpene 2 and cholesterol inhibited hemorrhagic activity of *B. jararaca* venom by 30%, while the monoterpene 1 inhibited 100% hemorrhage (Table 1).

Edema provoked by *B. jararaca* venom was also inhibited by the extracts of *P. brasiliense* (Figure 5B). Those prepared in dichloromethane (Figure 5B, group II) and ethyl acetate (Figure 5B, group III) inhibited edema by 22%, while extracts in hexane and hydroalcoholic solution inhibited by 15% and 2%, respectively (Figure 5B).

Mice injected with *B. jararaca* venom (20 μg/g) died in about 10 hours. Incubation of venom with derived products (80 μg/g) did not protect the animals against the lethal effects of *B. jararaca* venom (data not shown). When algal extracts alone, products or vehicle (DMSO or saline) were injected into animals, no hemorrhage, edema or death was seen. Therefore, extracts and derived products were not toxic to mice.

## Discussion

Envenomation resulting from snakebites is a particularly important public health problem in rural areas of tropical and subtropical countries [1,2]. In Brazil, according to the registers of the Brazilian Ministry of Health, about 30,000 victims are envenomated per year with hundreds of deaths [1,4,40,41]. However, these numbers are not accurate since snakebites often occur in areas where there is no health unit or hospital to record the case. Snakebite is a neglected tropical disease; hence, little attention has been given by the pharmaceutical industry, governments and academia to improve antivenom therapy.

In Brazil, antivenom is the only treatment available against snakebites, but it has some limitations and disadvantages. Therefore, alternative treatments, including natural products, have been investigated to complement or to replace antivenom therapy. Plant extracts have been used in folk medicine to treat or to ameliorate several conditions, including snakebites, and many scientific reports have proven their efficacy [21,42]. In contrast, the research on marine algae as inhibitors of toxic effects of snake venoms is limited. Previous results from our group have shown the inhibitory activity of plants, marine sponges and seaweed against snake venoms, but little is known about antivenom properties of algae such as *P. brasiliense* [28,43-45]. An initial study was conducted by Claudino *et al.* [34] analyzing the inhibitory action of *P. brasiliense* extract against the toxic effects of *L. muta* snake venom. Apart from antivenom activities, some biological and pharmacological properties have been found in these algae and their derived products, such as defense against marine herbivores and antiviral activity [31,32].

In the present work, we showed that *P. brasiliense* crude extracts – prepared in n-hexane (HEX), dichloromethane (DCM), ethyl acetate (ETA) and hydroalcoholic solution (HYD) – and three isolated products from HEX fractions (two halogenated monoterpenes and a cholesterol) inhibited *in vivo* and *in vitro* activities induced by *B. jararaca* venom. All the solvents employed are of increasing polarities, therefore they able to extract molecules derived from the secondary metabolism. The medium polarity solvent DCM inhibited most activities of *B. jararaca* venom whereas HYD, with the highest polarity solvent, recorded no inhibitory activity.

*B. jararaca* venom is rich in serine proteinases, metalloproteinases, phospholipases and phosphoesterases that affect the nervous system, blood coagulation, muscle tissue, red blood cells and other organs [46]. Furthermore, these enzymes are directly involved in local effects that are responsible for amputations and deformities as well as in systemic symptoms in victims [47]. *B. jararaca* venom induces coagulation, and thrombin-like enzymes participate in such action, leading plasma to clot and venom-induced consumptive coagulopathy. DCM, in particular, inhibited coagulation induced by *B. jararaca* venom, preventing fibrin production. However, it is unknown whether DCM extract interacts with components of the intrinsic and/or extrinsic pathways of the coagulation system. Moreover, DCM, HEX and ETA prevented proteolytic activity of *B. jararaca* venom, so that they may be interacting with proteases in such venom. In contrast, the extract prepared in HYD failed to inhibit proteolysis and coagulation provoked by *B. jararaca* venom. The two monoterpenes and the cholesterol fraction inhibited proteolysis of *B. jararaca* venom.

The crude extracts of *P. brasiliense* and the isolated products protected mice from hemorrhage and edema, except for HYD extract. The monoterpene 2 and the cholesterol derivatives inhibited around 30% of hemorrhage, whereas the monoterpene 1 fully inhibited it. Hemorrhagic activity of venoms is due to the action of metalloproteases and phospholipases A_2_ (PLA_2_). Several symptoms of envenomation by snakes such as coagulation and hemorrhage are associated with such enzymes, and they are one of the major groups of enzymes that cause the deleterious effects of venoms in victims [48,49]. The antivenom properties of extracts and derived products may be related to an inhibitory action upon such key enzymes. In the literature, many hypotheses have been proposed as mechanisms of inhibitory action of plants as well as any other natural sources, as protein precipitation, enzyme inactivation, metal chelation and antioxidant effect. However, the mechanism by which neutralization occurs is unknown [50].

In addition to hemorrhage, edematogenic activity should be considered in envenomation. It may be caused by the action of PLA_2_s that are poorly inhibited by commercial antivenoms [51]. PLA_2_s are calcium-dependent enzymes from venoms that induce edema, myotoxicity and lysis of the red blood cells, and these effects may contribute to morbidities or disabilities frequently observed in *B. jararaca* envenomation [52]. The DCM (98%) and HEX (60%) extracts effectively inhibited hemolysis. In the literature, researchers have made efforts in order to neutralize PLA_2_ enzymes, providing protection against snakebite symptoms. In this regard, edema induced by *B. jararaca* venom was inhibited by the extract of *P. brasiliense* as well in the present study. PLA_2_s also participate in the formation of lysolecithin (also called lysophosphatidylcholine), which is formed by the hydrolysis of phospholipids. Moreover, the toxic action of lysolecithin formed by the action of PLA_2_s from other sources of venoms has also been investigated. A PLA_2_ isolated from *L. muta* venom modulates natural killer activity by the protein kinase C pathway [53]. *B. jararaca* venom contains several isoenzymes of PLA_2_, serine proteases and metalloproteases that cause edema, coagulation and hemorrhage, respectively. However, any inhibitory effect cannot be attributed just to a specific group of enzymes since DCM and HEX extracts and monoterpene 1 fully inhibited all these toxic activities. It is worth noting that the less polar fractions, DCM and HEX, inhibited the toxic activities of *B. jararaca* venom more effectively, while the more polar, HYD did not inhibit such activities.

These products derived from *P. brasiliense* could be useful for treating the local effects induced by *B. jararaca* venom, since antivenom is not fully effective in preventing them, leading snakebite victims to amputations. To produce a single molecule or extract able to inhibit all the toxic activities of snake venom is a challenge as is developing an effective treatment, since there are ontogenetic variations in snake venom of *B. jararaca* [54]. *B. jararaca* venom, or any other snake venom, has different domain composition, glycosylation patterns and tertiary or quaternary structure, thus displaying different toxicity profiles and different or variable susceptibilities to inhibitors [55]. This fact greatly increases the difficulty in obtaining an entirely effective molecule able to fully neutralize the toxic effects and symptoms of envenomation by snakes. However, natural products such as the algal extracts described in the present paper provide hope for the future.

Marine seaweed produce biologically active molecules that have several pharmacological effects. In the present study, *P. brasiliense* extracts and three isolated products neutralized the main harmful effects of *B. jararaca* venom, both systemic (hemolysis, coagulation and hemorrhage) and local (edema and hemorrhage) symptoms observed in snakebite victims. However, venom-induced lethality was not prevented by algal extracts. In our opinion, the ineffectiveness concerning lethal outcomes should not be considered a discouraging result, since antivenom therapy efficiently neutralizes it [51]. In conclusion, these extracts have potential to be used to complement treatment of victims of *B. jararaca* envenomation. The present research aims to stimulate the development of new antivenom drugs based on further studies on algal and other natural products.

## Conclusion

The crude extracts of *P. brasiliense* and their isolated products inhibited *in vivo* and *in vitro* some harmful effects induced by the venom of *B. jararaca*. Therefore, these marine algae have biotechnological potential to lead to the development of alternative or complementary therapy for snake envenomation caused by *B. jararaca*.

### Ethics committee approval

The present study was approved by UFF Institutional Committee for Ethics in Animal Experimentation (CEUA, 200/10). All experiments were in accordance with the guidelines of the Brazilian Committee for Animal Experimentation (COBEA).
